# Retrieval practice enhances new learning: the forward effect of testing

**DOI:** 10.3389/fpsyg.2014.00286

**Published:** 2014-04-04

**Authors:** Bernhard Pastötter, Karl-Heinz T. Bäuml

**Affiliations:** Department of Experimental Psychology, Regensburg UniversityRegensburg, Germany

**Keywords:** learning, long-term memory, testing effect, retrieval practice, interference

## Abstract

In the last couple of years, there has been a dramatic increase in laboratory research examining the benefits of recall testing on long-term learning and retention. This work was largely on the backward effect of testing, which shows that retrieval practice on previously studied information, compared to restudy of the same material, renders the information more likely to be remembered in the future. Going beyond this prominent work, more recent laboratory research provided evidence that there is also a forward effect of testing, which shows that recall testing of previously studied information can enhance learning of subsequently presented new information. Here, we provide a review of research on this forward effect of testing. The review shows that the effect is a well replicated phenomenon in laboratory studies that has been observed for both veridical information and misinformation. In particular, the review demonstrates that the effect may be applied to educational and clinical settings, enhancing learning in students and reducing memory deficits in clinical populations. The review discusses current theoretical explanations of the forward effect of testing and provides suggestions for future research directions.

Retrieval practice enhances learning and long-term memory. Supporting such view, the results from numerous previous studies have shown that retrieval of previously studied information can increase its long-term retention more than repeated study or elaborative encoding of the information do (Karpicke and Roediger, 2008; Karpicke and Blunt, 2011). Although the effect has already been reported over 100 years ago (Abbott, 1909), it is only in the last couple of years that psychologists devoted a considerable amount of research to study this retrieval practice effect in more detail. This recent work has demonstrated that the effect is a robust and general phenomenon within lab-based studies in the memory literature (for reviews, see Roediger and Butler, 2011; Karpicke, 2012). It can be broadly applied, enhancing student learning in educational practice and reducing memory deficits in clinical patient groups (for reviews, see Middleton and Schwartz, 2012; Dunlosky et al., 2013).

The finding that retrieval practice of previously studied information can enhance its long-term retention is referred to as the backward effect of testing in the following and demonstrates what Roediger and Karpicke (2006) called a direct benefit of testing. However, there are also indirect benefits of testing. A particularly striking indirect benefit, referred to as the forward effect of testing in the following, is the finding that recall testing of previously studied information can increase long-term retention of subsequently studied new information. The forward effect of testing is particularly striking because it is on learning of information that is not necessarily related to the previously tested material. The present review provides a brief overview of research on the forward effect of testing. It will show that the forward effect is a well replicated phenomenon in laboratory studies that generalizes to different kinds of veridical information and misinformation. The review will further show that the effect can be applied to educational and clinical settings. Theoretical explanations of the effect will be discussed. Finally, the review will provide suggestions for future research directions and conclude by discussing other forms of retrieval than recall testing that may promote forward effects on new learning.

## Empirical evidence

The forward effect of testing has been shown to be a robust and replicable phenomenon in laboratory studies of memory research. Szpunar et al. (2008) observed the effect in multiple-list learning when using words as item material. Participants studied five word lists in anticipation of a final cumulative recall test. Prior to the experiment, participants were told to expect different activities that may follow the presentation of each single list: solving math problems, restudy of words from a just studied list, or immediate free recall of words from a just studied list. The experimenter pretended that activities following each list were determined randomly, whereas, in fact, interlist activities differed between experimental groups, and participants passed through the same activities, i.e., maths, restudy, or immediate recall testing, after study of lists 1–4 within each experimental group. Critically, all participants were tested immediately on the last list in the study sequence, referred to as target list 5. Two striking results emerged in this list 5 recall test: Participants who had been tested immediately on lists 1–4 recalled about twice as much list 5 items than the two non-tested groups; in addition, they showed much fewer prior-list intrusions than did participants in the two other groups. These results indicate a beneficial forward effect of recall testing in multiple-list learning. Because retrieval practice of lists 1–4 but not restudy of the lists affected list 5 recall, the results indicate a retrieval-specific effect.

The forward effect of testing in multiple-list learning has since been replicated in more recent laboratory work using different numbers of word lists and both related and unrelated words as item material (Pastötter et al., 2011; Nunes and Weinstein, 2012; Bäuml and Kliegl, 2013; see also Darley and Murdock, 1971; Tulving and Watkins, 1974). The effect is not restricted to the learning of words, but generalizes to the learning of various kinds of materials used in the memory laboratory context, including complex text (Wissman et al., 2011), narratives (Chan et al., 2009), pictures (Pastötter et al., 2013), videos (Szpunar et al., 2013), faces and names (Weinstein et al., 2011).

## Theoretical explanations

Both encoding and retrieval explanations have been put forth to account for the forward effect of testing on list learning in laboratory studies. Retrieval explanations typically assume that recall testing between the study of lists promotes contextual list segregation, which may enhance list differentiation and reduce interference between lists at test (Szpunar et al., 2008; Bäuml and Kliegl, 2013; see also Chan and McDermott, 2007; Brewer et al., 2010; Sahakyan and Hendricks, 2012). Specifically, it has been suggested that retrieval activities between the study of lists drive mental context change that can promote list segregation (Howard and Kahana, 2002; Jang and Huber, 2008). At test, improved list segregation then permits participants to use list-specific context cues and create more focused memory search, reducing interference from non-target lists. This retrieval explanation of the effect was recently supported by work analyzing response latencies of target items at test (Bäuml and Kliegl, 2013). In this laboratory study, participants studied three lists of words and were tested immediately on target list 3. Non-target lists 1 and 2 were either tested immediately or restudied. Both recall rates and response latencies of list 3 target items were analyzed. Recall testing of non-target lists not only enhanced list 3 recall rates but also reduced response latencies of list 3 target items. Because, in the memory laboratory context, reduced response latency is assumed to reflect a reduction in participants' memory search set size (Wixted and Rohrer, 1993; Rohrer and Wixted, 1994), the finding by Bäuml and Kliegl indicates that recall testing between the study of lists can induce more focused memory search on target items, reducing or even eliminating interference from non-target material at retrieval.

In contrast, encoding explanations of the forward effect of testing assume that recall testing of prior non-target materials improves encoding of the subsequently studied target material. Specifically, it has been suggested that testing induces a reset of the encoding process, making the encoding of the later lists as effective as the encoding of the earlier lists (Pastötter et al., 2011), or a change in participants' encoding strategy, enhancing elaborative encoding for the later lists compared to the earlier lists (Wissman et al., 2011). Recent neurocognitive work on human brain oscillations in the alpha frequency range (8–14 Hz) supports the reset-of-encoding view. Alpha power during item encoding increases with increasing study material, both within and across lists, a finding that has been attributed to impoverished item encoding due to memory load and inattention (Sederberg et al., 2006; Pastötter et al., 2008, 2011; Serruya et al., 2014). Crucially, recall testing between the study of lists has been shown to disrupt alpha power increases across lists, indicating that testing between the study of lists resets the encoding process for each single list (Pastötter et al., 2011; for similar results in related paradigms, see Pastötter et al., 2008, and Hanslmayr et al., 2012).

The forward effect of testing generalizes to different kinds of information. This provides good reason to believe that the effect is not restricted to the laboratory context, but generalizes to real educational learning environments. Of course, this remains to be shown in future work. Regarding theoretical explanations, however, generalization from the laboratory context to educational environments may not be entirely warranted. Because student learning in the classroom may differ from list learning in the laboratory in important aspects, additional or alternate factors to the ones suggested in the laboratory context may account for the effects of testing in educational environments. Possible candidates for such factors may include test expectancy, feedback, conceptual structuring of materials, or procedural rule learning.

## Testing in education

Testing can enhance student learning (Roediger et al., 2011; Roediger and Pyc, 2012; Dunlosky et al., 2013). Regarding the backward effect of testing, there is ample evidence that testing previously studied subject matter can increase its long-term retention. For instance, this has been shown in laboratory and classroom studies for the learning of foreign languages (Pyc and Rawson, 2010), statistics (Lyle and Crawford, 2011), medical knowledge (Larsen et al., 2009), and history facts (Carpenter et al., 2009), indicating that the backward effect of testing can be applied to educational practice. In contrast, only very recently a first step has been taken by Szpunar et al. (2013) in a laboratory study using educational materials to examine whether the forward effect of testing can be applied in an educational setting.

In this recent work, Szpunar et al. (2013) examined whether recall testing helps students learn the contents of an online video lecture in an introductory course in statistics. The video lecture was divided into four segments and students were asked to study the contents of each segment in anticipation of a final cumulative recall test. Segment 4 was always tested immediately by asking students knowledge questions about key concepts from this part of the video. Segments 1–3 were either also tested immediately, restudied by showing the questions along with the answers, or followed by a mathematical distractor after each single segment. The results in the immediate segment 4 recall test showed that prior testing of sections 1–3 increased the number of correctly answered segment 4 questions and reduced source confusions, indicating a forward effect of testing in student learning. Consistent with the reset-of-encoding view, the results further showed that recall testing helped students sustain high attention to encoding from early to late lecture content, by encouraging task-relevant activities like note taking and discouraging task-irrelevant activities like mind wandering. In addition, the recall testing reduced both test anxiety and subjectively experienced mental effort. Together, these findings demonstrate that the forward effect of testing can enhance student learning in an educational setting.

Following Szpunar et al. (2013), future laboratory and classroom studies may examine whether the forward effect of testing generalizes to other contents and other learning environments in and outside the laboratory. Moreover, future work may investigate whether and how students of different ages and students with specific memory or attention deficits can benefit from testing in education.

## Testing in clinical populations

Testing can enhance learning in clinical populations (Wilson, 2009). Regarding the backward effect of testing, testing of previously studied information has been shown to increase its long-term retention in persons with Alzheimer's disease (Camp et al., 1999; Small, 2012), multiple sclerosis (MS; Sumowski et al., 2010a, 2013), and traumatic brain injury (TBI; Sumowski et al., 2010b, 2014), indicating that the backward effect is broadly present in clinical populations. In contrast, regarding the forward effect of testing, only very recently Pastötter et al. (2013) were the first to investigate whether retrieval enhances new learning in a clinical subject sample, examining the effects of recall testing in persons with severe TBI.

In their clinical laboratory study, Pastötter et al. (2013) examined both backward and forward effects of testing in persons with severe TBI, in comparison to healthy controls. Participants studied three lists of items, which were pictures of everyday things presented together with their names. They were asked to remember the items for a final cumulative recall test. All participants were tested immediately on list 3. In the testing condition participants were also tested immediately on lists 1 and 2, whereas in the distractor condition they counted backwards in steps of ones after study of lists 1 and 2. The results showed that testing effects were not restricted to healthy participants. Instead, both the backward and the forward effect of testing were equally present in persons with severe TBI and healthy controls. Indeed, regarding the forward effect in the immediate list 3 recall test, recall testing of lists 1 and 2 improved list 3 recall and reduced prior-list intrusions in persons with TBI to the same degree as in healthy controls. Apparently, testing can largely reduce memory deficits and enhance learning in persons with severe TBI. Elaborating on the generalizability of the forward effect to other clinical populations is a high priority for future work.

In prior work, memory deficits in persons with TBI, MS, and Alzheimer's disease have been suggested to arise mainly from deficient encoding, and less from deficient retrieval (Greene et al., 1996; DeLuca et al., 2000, 2013; Blanchet et al., 2009). On the basis of Pastötter et al.'s (2011) study which showed that alpha oscillations can be a marker of encoding efficacy in multiple-list learning, future work may measure oscillatory brain activity in persons with TBI, MS, and Alzheimer's disease to examine the degree to which testing can reduce encoding deficits in different clinical populations. Complementing such work, future studies may also address patients' retrieval abilities. Following Bäuml and Kliegl (2013), for instance, response latency analysis may be employed to examine whether recall testing induces more focused search on subsequently studied items, a finding reported in healthy persons.

## Testing before misinformation

Testing can enhance learning of misinformation (Chan et al., 2009). This has been shown in the misinformation paradigm (Loftus et al., 1978). In this paradigm, participants witness an event, for instance by watching a video of a crime scene, and next are exposed to a narrative description of the event that contains misinformation on specific detail (e.g., the witness is told that the bad guy drove off with a red car; however, the car in the video was actually blue). At test, participants are asked to recall the details of the witnessed event in the video. The typical finding is that the previous presentation of misinformation impairs memory for the details of the original event, indicating that eyewitnesses' memories are malleable and can be influenced by exposure to subsequently presented misinformation.

Examining the effects of testing in the misinformation paradigm, recent laboratory work by Chan and colleagues has shown that recall testing between the encoding of the event and the encoding of the misinformation can increase participants' suggestibility to the misinformation on a final recall test (Chan et al., 2009, 2012; Chan and Langley, 2011; Chan and LaPaglia, 2013; Gordon and Thomas, 2014; Wilford et al., 2014). For instance, Chan et al. (2009) let participants watch a video clip of a terrorist attack. After watching the video, participants either took an immediate cued-recall test on specific details about the video or completed an unrelated distractor task. After that, all participants listened to an audio narrative that described the video, without being warned that the narrative contains misinformation. Finally, participants took a final cued-recall test that was identical to the immediate recall test. The results in the final recall test showed that immediate testing enhanced incorrect recall of misinformation, indicating that immediate testing makes witnesses susceptible to misinformation.

The finding by Chan et al. (2009) seems remarkable, because it is in direct contrast to what the backward effect of testing predicts. According to this effect, immediate testing should have enhanced memory for the witnessed event and thus reduced suggestibility to misinformation. This is not what the results showed. The finding, however, is perfectly in line with what the forward effect of testing predicts. According to this effect, immediate testing enhances encoding of the subsequently presented misinformation and thus increases suggestibility to the misinformation on the final recall test. Such an encoding view on the misinformation effect is in line with current encoding explanations of the forward effect of testing (Pastötter et al., 2011; Wissman et al., 2011), and is also well supported by more recent research on the effects of testing in the misinformation paradigm (e.g., Chan and Langley, 2011; Gordon and Thomas, 2014; Wilford et al., 2014). The generalizability of laboratory effects to real-life scenarios needs to be tested.

## Concluding remarks

The review of the existing literature on the forward effect of testing indicates that, within the lab-based studies in the memory literature, the effect is a replicable phenomenon. There is also evidence that, just like the backward effect of testing, the forward effect of testing may be applied to educational and clinical practice, showing that recall testing can enhance student learning and reduce learning deficits in people with severe TBI. Research further showed that the effect pertains to both veridical information and misinformation. Thus, the existing literature on the forward effect of testing already provides important insights into how recall testing can affect learning and memory.

Further important research questions should be addressed in the future. First, the prior laboratory work used recall tests both in the immediate and the final test phases, and future work may rather use multiple choice, short answer, or recognition testing to examine whether the effect generalizes to other test formats more often used in educational practice. Second, following the laboratory study with educational materials by Szpunar et al. (2013), classroom studies may examine the forward effect of testing in real educational environments. Third, following Pastötter et al.'s (2013) study on persons with TBI, future work on the generalizability of the forward effect of testing to different clinical populations is eligible. Fourth, laboratory work showed that different forms of retrieval—e.g., episodic memory retrieval of studied item lists, semantic memory retrieval of general knowledge facts, and autobiographical memory retrieval of preexperimental context—, can promote contextual list segregation and enhance learning (e.g., Pastötter et al., 2008, 2011; see also Howard and Kahana, 2002; Jang and Huber, 2008). Therefore, discovering exactly what forms of retrieval and what processes at retrieval promote the forward effect of testing is a high priority for future work.

### Conflict of interest statement

The authors declare that the research was conducted in the absence of any commercial or financial relationships that could be construed as a potential conflict of interest.
